# Cerebrospinal fluid and blood exosomes as biomarkers for amyotrophic lateral sclerosis; a systematic review

**DOI:** 10.1186/s13000-024-01473-6

**Published:** 2024-03-01

**Authors:** Shahram Darabi, Armin Ariaei, Auob Rustamzadeh, Dariush Afshari, Enam Alhagh Charkhat Gorgich, Leila Darabi

**Affiliations:** 1 Cellular and Molecular Research Center, Research Institute for Non-communicable Diseases, Qazvin University of Medical Sciences, Qazvin, Iran; 2 Student Research Committee, Faculty of Medicine, Iran University of Medical Sciences, Tehran, Iran; 3 Department of Anatomical Sciences, School of Medicine, Iran University of Medical Sciences, Hemmat Highway, next to Milad Tower, Tehran, Iran; 4 Department of Neurology, School of Medicine, Kermanshah University of Medical Sciences, Kermanshah, Iran; 5 Department of Anatomy, School of Medicine, Iranshahr University of Medical Sciences, Iranshahr, Iran; 6 Department of Neurology, Tehran Medical Science Branch, Amir Al Momenin Hospital, Islamic Azad University, Tehran, Iran

**Keywords:** Amyotrophic lateral sclerosis, Biomarkers, Extracellular vesicles, Exosomes

## Abstract

**Background:**

Amyotrophic lateral sclerosis (ALS) is a progressive and fatal motor neuron disease. Due to the limited knowledge about potential biomarkers that help in early diagnosis and monitoring disease progression, today’s diagnoses are based on ruling out other diseases, neurography, and electromyography examination, which takes a time-consuming procedure.

**Methods:**

PubMed, ScienceDirect, and Web of Science were explored to extract articles published from January 2015 to June 2023. In the searching strategy following keywords were included; amyotrophic lateral sclerosis, biomarkers, cerebrospinal fluid, serum, and plama.

**Results:**

A total number of 6 studies describing fluid-based exosomal biomarkers were included in this study. Aggregated proteins including SOD1, TDP-43, pTDP-43, and FUS could be detected in the microvesicles (MVs). Moreover, TDP-43 and NFL extracted from plasma exosomes could be used as prognostic biomarkers. Also, downregulated miR-27a-3p detected through exoEasy Maxi and exoQuick Kit in the plasma could be measured as a diagnostic biomarker. Eventually, the upregulated level of CORO1A could be used to monitor disease progression.

**Conclusion:**

Based on the results, each biomarker alone is insufficient to evaluate ALS. CNS-derived exosomes contain multiple ALS-related biomarkers (SOD1, TDP-43, pTDP-43, FUS, and miRNAs) that are detectable in cerebrospinal fluid and blood is a proper alternation. Exosome detecting kits listed as exoEasy, ExoQuick, Exo-spin, ME kit, ExoQuick Plus, and Exo-Flow, are helpful to reach this purpose.

## Introduction

Amyotrophic lateral sclerosis (ALS) is a complex fatal neurodegenerative disease characterized by the progressive degeneration of upper motor neurons (UMNs) and lower motor neurons (LMNs) in different areas of the brain and spinal cord [1–3], eventuating in muscle paralysis, atrophy, weakness, respiratory failure, and lastly death within 3–5 years of disease onset [4]. According to study reports, 223,000 people worldwide were affected by ALS, and estimated that this number will increase by 69% in 2040, primarily due to the aging population [5]. As this fatal disease is expected to spread over time and affect more individuals, improving our understanding of ALS seems vital. ALS is described in two types: Familial ALS (fALS) and sporadic ALS (sALS). The latter, with no specific inheritance pattern, accounts for nearly 90% of all ALS cases, whereas fALS accounts for almost 5–10% of the ALS population [4].

When ALS symptoms initially appear in the limbs, leading to movement or walking difficulties, it is called limb-onset ALS. Another presentation of ALS, which is more progressive than limb-onset ALS [6], is classified as bulbar-onset ALS, when symptoms occur in the face or neck, leading to difficulties in swallowing or speech [6, 7]. Although fALS severity is higher than sALS, both types are clinically identical [5]. Clinical diagnosis of ALS is usually tricky due to its overlapping symptoms with other neurological disorders and the lack of a specific diagnostic test [8]. To date, due to ALS’s complex nature and limited knowledge of the underlying mechanisms that cause it, there is no treatment. Existing strategies are based only on disease management, survival enhancement, and symptom therapy [9]. Therefore, the discovery and development of strategies that can help in early diagnosis, demonstrate target engagement, monitor the course of the disease, and serve as an indicator of treatment efficacy seems vital for ALS patients [5, 10, 11]. To achieve these goals, biomarkers are a suitable option.

Currently, the ALS pathogenic mechanisms are still largely unknown [12], however, possible mechanisms may include mitochondrial dysfunction, the mutant SOD1 effects, and glutamate excitotoxicity [7, 12–14]. In addition, several mutant genes associated with ALS have been identified [5, 7]. For instance, in 1993, due to the discovery by a group of scientists supported by the National Institute of Neurological Disorders and Stroke, the mutant *SOD1* gene was associated with some fALS patients [15]. Afterward, other additional genetic mutations have been in ALS like TDP-43, [16] which is primarily responsible for protein synthesis and RNA processing [16, 17]. These pathogenic proteins have been observed to accumulate in the plasma-derived and central nervous system (CNS)-derived extracellular vesicles and act as diagnostic biomarkers [18]. Attempts to discover fluid-based ALS biomarkers have been accomplished chiefly using CSF due to its close position to the neuroanatomical region affected by the disease. However, recent findings have also studied serum and plasma, and scientists’ attempt to conduct studies based on urine and saliva is emerging [10, 19]. For instance, cystatin C, detected in the CSF samples of ALS patients, also known as a cysteine protease inhibitor is conceptualized to participate in the procedures resulting in the formation of the Bunina bodies in the intraneuronal areas [10]. In addition to mutant gene products, bio-fluids contain neurofilament proteins, inflammatory mediators, and metabolic markers that provide information about ALS progression.

In this review, we aim to investigate relevant findings about ALS diagnostic fluid-based biomarkers by introducing the potential ALS-associated proteins or factors accumulated in the exosomes of blood and cerebrospinal fluid (CSF), whether as a result of gene mutations or other causes. Improving our knowledge of ALS diagnosis can get us closer to developing novel yet definitive ALS therapeutics in the future.

## Materials and methods

This study was conducted based on the Preferred Reporting Items for Systematic Reviews and Meta-Analyses (PRISMA) guidance [11, 12]. Moreover, the protocol of the study was submitted in the PROSPERO website.

### Eligibility criteria

In this study, retrospective, prospective, and cross-sectional studies published in peer-reviewed journals with the aim of investigating exosomal biomarkers in the ALS disease were included. Also, studies with less than 10 participants classified as case reports and case series were excluded. Moreover, there was a publication year restriction, in which only novel biomarkers described from articles published since 2015 were included. Finally, studies published as a conference proceedings were not eligible for this study. In this study, biomarkers as a prediction, diagnostic, or prognosis measured in the fluid samples, including serum/plasma and CSF, were included.

### Search strategy and databases

Three databases (PubMed, ScienceDirect, and Web of Science) were explored to extract articles with publication years ranging from January 2015 to June 2023. In the searching strategy following keywords were considered; amyotrophic lateral sclerosis, biomarkers, cerebrospinal fluid, serum, and plasma. Eventually, the following search strings were utilized to find relevant articles from PubMed, ScienceDirect, and Web of Science databases.

• In the PubMed database, based on the purpose of the study and keywords following search string was built: ((amyotrophic lateral sclerosis [Title/Abstract] AND biomarker[Title/Abstract]) AND exosomes[Title/Abstract] AND (CSF[Title/Abstract] OR serum[Title/Abstract] OR plasma[Title/Abstract]) NOT (spinal cord injury)))
• In Web of Science and ScienceDirect databases articles were extracted based on the following search string: ((amyotrophic lateral sclerosis AND biomarker AND exosomes) AND (CSF OR serum OR plasma) NOT (spinal cord injury))

### Study selection

Conference abstracts, case reports, and case series were omitted in advance of initial screening. Evaluating the title and abstract of the extracted articles were implemented through three reviewers (AA, SD, and DA). In the evaluation of the title and abstract, the presence of relevant keywords was considered. Subsequently, the methodology of the remaining articles was evaluated from multiple aspects, including statistical analysis, sample size, and the tool by which biomarkers were assessed. If the article’s full text were not free to access, a request would be made to obtain the full text. If the request was responded to without a positive outcome, the manuscript was excluded from further evaluation.

### Data extraction

The required data from the inputs were extracted by one of the researchers (AA) into a table, in which assessed tools and the cutoff point for diagnostic accuracy were extracted. It is worth mentioning that only confirmed biomarkers detected in serum/plasma or CSF were reported in the tables. Other biomarkers were mentioned in the main text.

### Quality assessment

The Critical appraisal (CA) test as a questionnaire assessment tool to evaluate the risk of bias in the selected studies was utilized with the judgment of two of the reviewers (AR and AA). Moreover, the included studies were assessed based on the criteria discussed in the Standards for Reporting of Diagnostic Accuracy (STARD) checklist. Based on STARD checklists included studies should contain a diagnostic accuracy test (including predictive values, specificity, sensitivity, or area under the curve), enrollment of patients with valid criteria (El Escorial criteria), and report the statistical results with their precision.

## Results

**Fig. 1:**
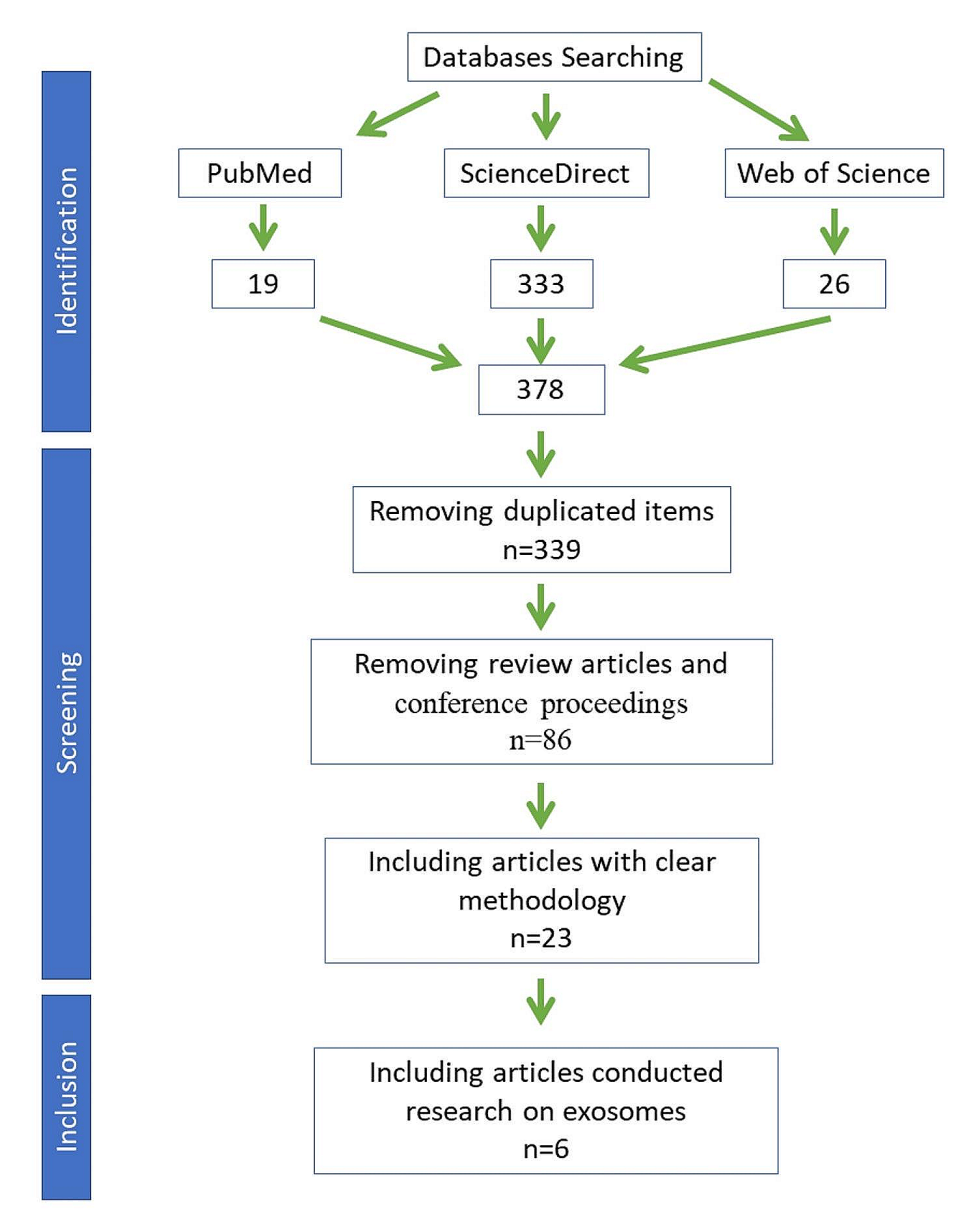
PRISMA flow diagram of the included studies

In the initial searching, 378 inputs were obtained from three databases (PubMed, ScienceDirect, and Web of Science). After removing duplicated inputs and applying inclusion criteria, 6 original studies reported exosomal fluid-based biomarkers were included in this study (Fig. 1).

### Extracellular vesicles (EVs)

Nearly all cells [20], including the nervous system cells, are observed to release 30 to 2000 nm diameter vesicles to the extracellular space [21]. Being found in physiological fluids, blood-derived EVs and CNS-derived EVs cross the blood-brain barrier (BBB) and are readily detectable in blood, CSF, and urine. Besides, these vesicles can be isolated from blood, urine, or CSF by several minimally invasive techniques. Since the secretion of macromolecules, including mRNAs, miRNAs, lipids, and proteins, is mediated by EVs [3, 21, 22] carrying specific biochemical signals [23], they provide us with precious information about cell status, especially during disease conditions. Two main EV types have been described as exosomes and microvesicles (MVs). MVs of ALS patients have been identified to be enriched by the accumulation of ALS-related proteins, namely SOD1, TDP-43, pTDP-43, and FUS [16], suggesting EV-mediated prion-like propagation of ALS disease [19], these mutant proteins retrieved in EVs are delivered across the brain cells and spread the disease (Table 1). In addition, the Chen et al. 2020 results investigating TDP-43, NFL, and pNFL in the exosomes derived from plasma samples reported an enhancement of TDP-43 and NFL in exosomes of the ALS group, over time highlighted its prognostic role [24]. Furthermore, exosomes are reported to transport ALS-specific downregulated miRNA biomarkers, including miR-27a-3p which could be detected in patients’ plasma through exoEasy Maxi and exoQuick Kit for exosome isolation and qRT-PCR method for miRNA detection [11, 25]. Also, the CORO1A protein could be detected in the exomes extracted from ALS patients’ plasma. It was suggested as a novel fluid biomarker highlighted by an upregulation that was highly correlated with disease progression [26] (Table 1).

**Table 1:** The plasma/serum biomarkers of amyotrophic lateral sclerosis disease

Biomarkers	Sample size	Method	Role	Highlight	Cutoff point	Area under curve (AUC)	Reference
Non exosomal biomarker							
miRNA-206	ALS: 27 Control: 13	miRCURY RNA Isolation Kit Biofluids (Exiqon Cat #EX300112) ExiLENT SYBR^R^ Green master mix (Exiqon Cat #203,421) and Exiqon microRNAs specific primers	Transcriptional regulation of gene expression	Increased value was correlated with a better prognosis	≤ 145.1 copies/200 µl serum	0.9917	Dobrowolny et al. 2021 [49]
miR-133a	ALS: 27 Control: 13	miRCURY RNA Isolation Kit Biofluids (Exiqon Cat #EX300112) ExiLENT SYBR^R^ Green master mix (Exiqon Cat #203,421) and Exiqon microRNAs specific primers	Transcriptional regulation of gene expression	Increased value was correlated with a better prognosis	≤ 108.2 copies/200 µl serum	0.8088	Dobrowolny et al. 2021 [49]
miR-151a-5p	ALS: 27 Control: 13	miRCURY RNA Isolation Kit Biofluids (Exiqon Cat #EX300112) ExiLENT SYBR^R^ Green master mix (Exiqon Cat #203,421) and Exiqon microRNAs specific primers	Transcriptional regulation of gene expression	Increased value was correlated with a better prognosis	≤ 11,183 copies/200 µl serum	0.8846	Dobrowolny et al. 2021 [49]
NFL	ALS: 30 Control: 20	The human NF-light assay (Cat No: 102,258) kits	Axonal transport and shaping cells	Significantly higher levels of serum NFL in patients with ALS and correlated with disease progression: 63.3 (46.9–98.1) pg/mL than in healthy controls: 5.3 (4.5–7.1) pg/mL	14.3 pg/mL	1	Sugimoto et al. 2020 [58]
CD14	ALS: 100 Control: 60	R&D Systems® ELISA Kits	An inflammatory marker secreted from Hepatocytes	Elevated in ALS compared to normal participants (ALS mean ~ 3.2 µg/mL, normal ~ 2.6 µg/mL)	2.73 µg/ml	0.93	Beers DR et al. 2020 [68]
C reactive protein	ALS: 100 Control: 60	R&D Systems® ELISA Kits	An inflammatory marker secreted from Hepatocytes	Elevated in ALS compared to normal participants (ALS mean ~ 5 µg/mL, normal ~ 1 µg/mL)	4.53 µg/ml	0.965	Beers DR et al. 2020 [68]
Lipopolysaccharide binding protein	ALS: 100 Control: 60	R&D Systems® ELISA Kits	Inflammatory markers secreted from Hepatocytes	Elevated in ALS compared to normal participants (ALS mean ~ 40 µg/mL, normal ~ 20 µg/mL)	40.8 µg/ml	0.966	Beers DR et al. 2020 [68]
Exosomes Biomarkers							
miR-146a-5p, miR-199a-3p, miR-151a-3p, miR-151a-5p, and miR-199a-5p (exosomes)	ALS: 20 Control: 20	ExoRNeasy Serum/Plasma Kit (cat. no. 77,023, Qiagen, Hilden, Germany) LightCycler® 480 Real-Time PCR System (Roche, Basel, Switzerland)	Not mentioned	Upregulated in ALS patients compared to control participants based on 2^−(ΔΔCt)^	Not mentioned	Not mentioned	Banack et al., 2020 [52]
miR-4454, miR-10b-5p, and miR-29b-3p (exosomes)	ALS: 20 Control: 20	ExoRNeasy Serum/Plasma Kit (cat. no. 77,023, Qiagen, Hilden, Germany) LightCycler® 480 Real-Time PCR System (Roche, Basel, Switzerland)	Not mentioned	Downregulated in ALS patients compared to control participants based on 2^−(ΔΔCt)^	Not mentioned	Not mentioned	Banack et al., 2020 [52]
miR-27a-3p (exosomes)	ALS: 10 Control: 20	ExoEasy Maxi, exoQuick Kit, and qRT-PCR	Not mentioned	Downregulated in ALS patients compared to control participants	Not mentioned	Not mentioned	Xu et al. 2018 [85]
CORO1A (exosomes)	ALS: 30 Control: 33	Sandwich enzyme-linked immunoassay (ELISA) (05657, Y-s Biotechnology, China)	Blocks autophagic flux and fusion with lysosomes	Increased 5.3-fold higher in ALS compared to controls. Increased with disease progression	Not mentioned	Not mentioned	Zhou et al. 2022 [26]
TDP-43 (exosomes)	ALS: 18	EXObuffer (Biovesicle Inc.)	RNA regulation	An increase was observed in TDP-43 over time	Not mentioned	Not mentioned	Chen et al. 2020 [24]
NFL (exosomes)	ALS: 18	Biotin-labeled anti-NFL monoclonal antibody (IBL, Hamburg, Germany)	Axonal transport and shaping cells	The concentration was increased in the rapid progression ALS group	Not mentioned	Not mentioned	Chen et al. 2020 [24]

### Superoxide dismutase 1 (SOD1)

Superoxide dismutase 1 (SOD1) is an abundant cytosolic and mitochondrial enzyme responsible for the clearance of superoxide molecules by breaking down superoxide radicals. As mentioned above, SOD1 was the first ALS-related protein to be detected in extracellular vesicles (EVs). Misfolded disulfide-cross-linked SOD1 aggregates have been observed in EVs from spinal motor neurons and glial cells of familial ALS (fALS) and some proportion of sporadic (sALS) cases [15]. It is noteworthy that in some other fALS and sALS cases, instead of mutations in SOD1, wild-type SOD1 has been observed [27]. As Sibilla et al. propose, SOD1 can self-replicate in vitro and transfer aggregates in culture [28]. Further, the aggregates of mutant SOD1 in EVs are associated with the prion-like propagation of the ALS pathology in the CNS [16]. This term is applied to diseases including Alzheimer’s disease (AD), Parkinson’s disease (PD), and Huntington’s disease (HD) that are caused by the deposition of several misfolded proteins. Moreover, by measuring SOD1 concentration in CSF we perceive an elevation in the values versus healthy controls [29].

### RNA-binding protein (FUS)

RNA-binding protein FUS is involved in DNA transcription, protein synthesis, RNA metabolism, stress granules formation resulting in neural death [30], and disarrangement of the splicing phenomenon [31]. Abnormal FUS protein aggregates have been observed in several neurodegenerative diseases, including ALS, FTLD, and polyglutamine disease [30], FUS aggregation in ALS appears at the early stages of the disease, contributing well to the disease pathogenesis [32] Compared to SOD1 or TDP-43 mutation carriers, FUS mutation carriers have shown a slightly younger age of disease onset and their survival time appeared to be shorter [33]. Similar to the TDP-43 protein, the mouse model expressing the human FUS protein has a short lifespan and exhibits an aggressive phenotype [34]. It is proposed that the FUS protein aggregations within stress granules might be associated with disease initiation [35]. However, the exact roles of FUS and stress granules in the pathogenesis of ALS and FTLD remain unclear [34]. Attempts to identify FUS-based biomarkers in plasma or CSF might offer developments in the therapy of inherited FUS-linked ALS [30].

### TAR DNA-binding protein 43 (TDP-43)

**Fig. 2:**
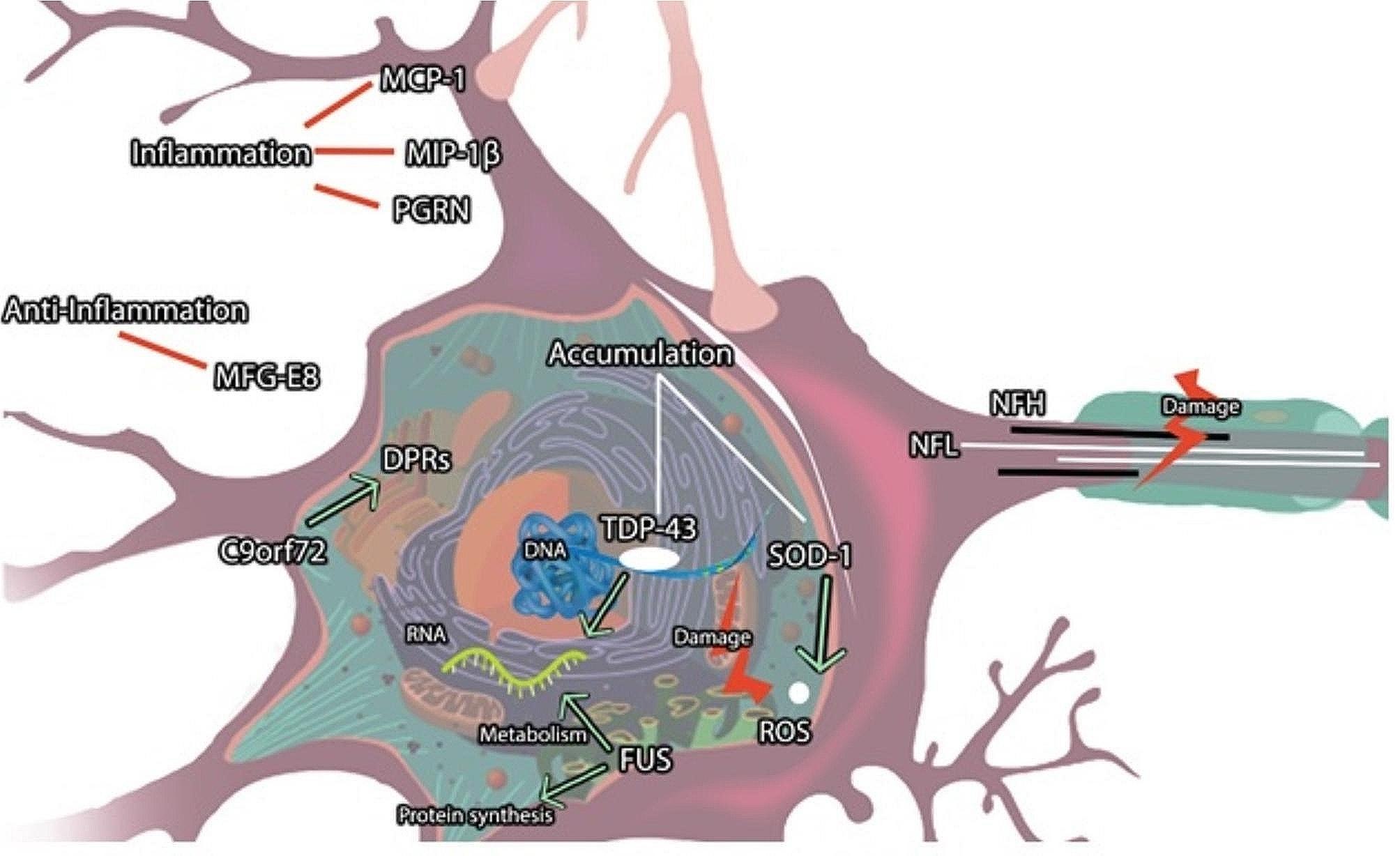
The ALS disease pathological signaling pathway. MFG-E8: milk fat globule-EGF factor 8, PGRN: Progranulin, MCP-1: macrophage chemoattractant protein-1, MIP-1β: macrophage inflammatory protein-1β, ROS: reactive oxygen species, SOD1: Superoxide dismutase 1, FUS: Fused in sarcoma, TDP-43: TAR DNA-binding protein 43, NFL: neurofilament light, NFH: neurofilament heavy, DPRs: dipeptide repeat proteins

**Fig. 3:**
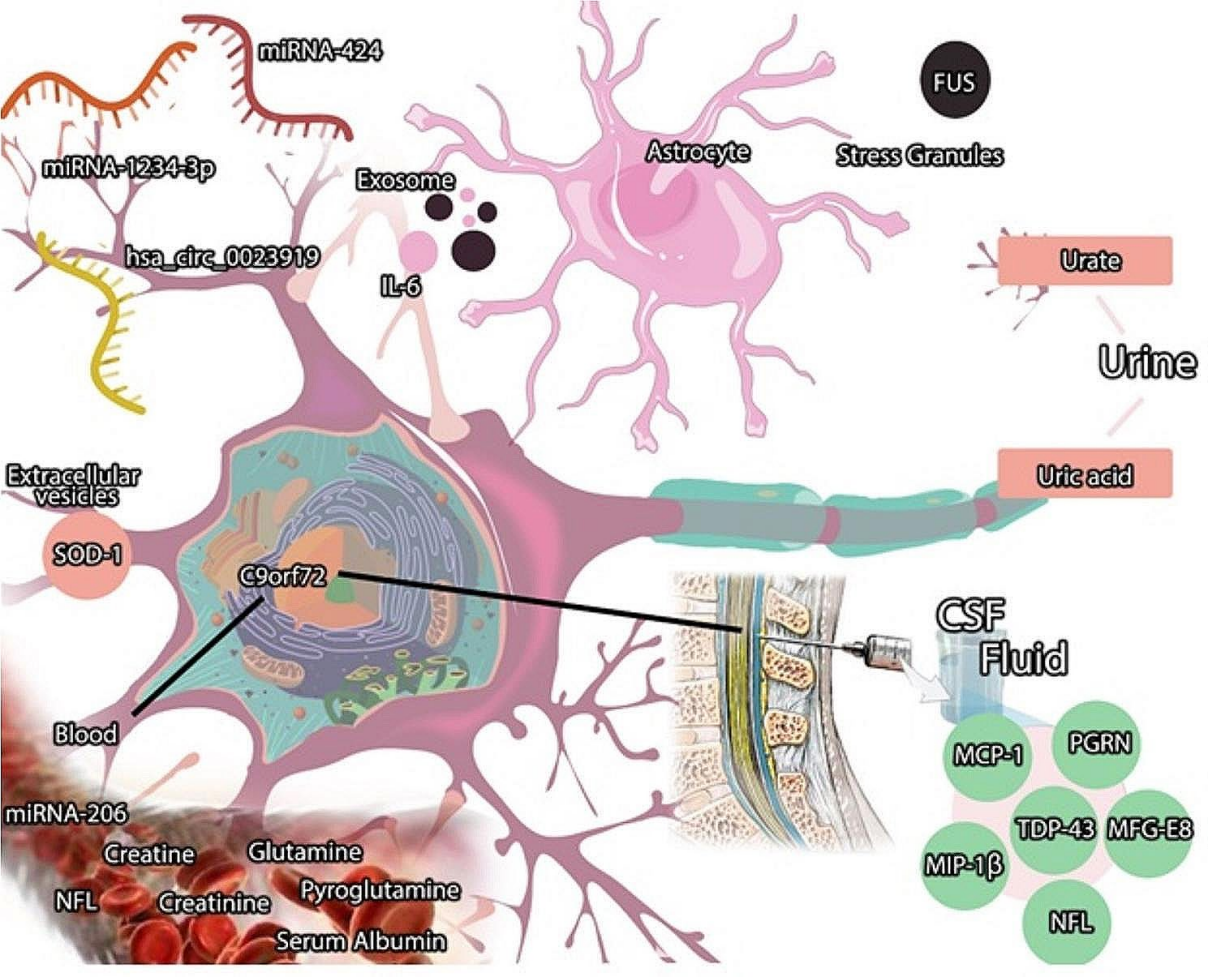
The ALS disease biomarkers in the CSF, Blood, Exomes, Extracellular vesicles, and Stress granules. Although, still there is no available method to detect C9orf72, based on in vitro and animal research this biomarker has the potential to be measured in the blood and CSF. CSF: cerebrospinal fluid, MFG-E8: milk fat globule-EGF factor 8, PGRN: Progranulin, IL-6: interleukin-6, MCP-1: macrophage chemoattractant protein-1, MIP-1β: macrophage inflammatory protein-1β, SOD1: Superoxide dismutase 1, FUS: Fused in sarcoma, TDP-43: TAR DNA-binding protein 43, NFL: neurofilament light, miRNA: micro RNA

Another protein retrieved in EVs is TAR DNA-binding protein 43 (TDP-43) and phosphorylated-TDP-43 (pTDP-43) [36]. The pathological hallmark underlying most ALS cases and half of the cases of frontotemporal lobar degeneration (FTLD) is the deposition of nuclear and cytoplasmic inclusions of (TDP-43), which regulates translational regulation, pre-mRNA splicing, and transcriptional repression [16, 37, 38]. Similar to SOD1, TDP-43 presents a prion-like structure, appearing misfolded in extracellular fluids (Fig. 2). Plasma-derived EVs in familial types of ALS have been observed to be enriched with mutant TDP-43 aggregates; however, the serum has not shown the same result [39]. Furthermore, TDP-43 accumulations have been found in the EV fraction of CSF from ALS and Frontotemporal dementia (FTD) [21]. Similar to TDP-43, many other mutant genes provide ALS degenerative conditions that may resemble FTD [16]; thus, biomarkers should be examined carefully (Fig. 3).

### MicroRNA (miRNA)

As it was previously mentioned, miR-27a-3p can be detected in the exosomes of ALS patients. However, there are a wide range of miRNAs in which some proportion of them can be detected in exosome, while others are presented as circulating biomarkers in the serum/plasma or CSF [40–43]. miRNAs are small, non-coding RNA molecules playing roles as endogenous transcriptional regulators of gene expression. Being present in human physiological fluids, miRNAs are considered promising biomarkers for neurodegenerative diseases like PD, AD, HD, and ALS [44]. Detectable changes in many serum miRNA levels have been observed, of which miR-206 seems to be very promising as its higher expression level in ALS patients has been confirmed by many studies [45–48]. However, the miR-206 circulating level has been reported to decrease along the disease progression [45, 49]. Moreover, considering different stages of ALS, some miRNAs are involved in apoptosis and the neurodegenerative phase of the disease, including miR-338, miR-142, and miR-183, whereas miR-206, miR-133a, and miR-133b associate with the muscular atrophy [50]. (Table 1) The serum level of all these miRNAs increases during the disease progression, except for miR-183 [50]. Although these biomarkers seem to represent a valid fluid-based biomarker for ALS, further investigation might still be required, as these changes in miRNAs might appear in other neurodegenerative conditions, particularly ALS-mimic disorders, and thus not specific to ALS pathogenesis. Apart from miRNAs, different types of RNAs, including lncRNAs, and circRNAs, all detectable in blood, may also represent other novel biomarkers for ALS [47] as they have already been proposed for some neurodegenerative diseases and cancers [51]. For example, an elevated level of circRNA hsa_circ_0023919 in blood samples [43, 47], as well as CSF [39] of SALS patients, has been observed, suggesting the potential utility of other types of RNAs. Moreover, in a cohort study conducted by Banack et al., the plasma samples were obtained from ALS and normal participants to determine the level of miRNAs in the neural-enriched EVs. In this research, first, the plasma was acquired from participants, subsequently, EVs were derived from the plasma, and the level of miRNAs was determined by the real-time PCR. The result of the study concluded in five upregulated (miR-146a-5p, miR-199a-3p, miR-151a-3p, miR-151a-5p, and miR-199a-5p) and three downregulated (miR-4454, miR-10b-5p, and miR-29b-3p) miRNAs [52].

### Non-exosomal fluid-based biomarkers

Levels of the NFH and NFL have been reported to be significantly elevated in the CSF of ALS than in healthy controls and patients without parenchymal CNS disease, indicating axonal impairment in ALS [8, 53, 54] With regards to blood, NFL has appeared in serum-derived EVs with a validity of 84–100% and specificity of 76–97%, whereas the data for NFH in serum has been 61–80% for validity and 72.1–83.7% for specificity, implying that serum NFH seems to be slightly a less potent biomarker [55]. Notably, the rise of NFL levels has been detected in several other CNS diseases, for example, in subarachnoid hemorrhage [56, 57], and thus is not specific to ALS. However, NFL and NFH are excellent biomarkers in differentiating ALS patients from healthy ones and ALS mimic diseases, based on the Sugimoto et al. 2020 study with a sensitivity and specificity of 100% [58] (Tables 1 and 2).

**Table 2:** The cerebrospinal fluid biomarkers of amyotrophic lateral sclerosis disease

Biomarkers	Sample size	Method	Role	Highlight	Cutoff point	Area under curve (AUC)	Reference
Non exosomal biomarker							
TDP-43	ALS: 36 Control: 24	Western blot (Thermo Fisher Scientific, Waltham, Catalog# 711,051) And immuno-infrared sensor	RNA regulation	89% Sensitivity and 83% Specificity for ALS versus healthy controls ALS: 1639.5 ± 2.6 cm^− 1^ SD Normal: 1646 ± 5.5 cm^− 1^ SD	1643 cm^− 1^ (Immuno-infrared sensor)	0.93	Beyer et al. 2021 [38]
MCP-1	ALS: 77 Control: 13	Bio-PlexProTM commercial kit (Bio-Rad Laboratories, Inc., Hercules, CA, USA)	Chemokine	No correlation between duration and the clinical stages of the disease MCP-1 level increased (234.89 pg/mL vs. 160.95 pg/mL, *P* = 0.011)	Not mentioned	Not mentioned	Martínez et al. 2020 [66]
MIP-1β	ALS: 77 Control: 13	Bio-PlexProTM commercial kit (Bio-Rad Laboratories, Inc., Hercules, CA, USA)	Chemokine	No correlation between duration and the clinical stages of the disease The MIP-1β level increased (10.68 pg/mL vs. 4.69 pg/mL, *P* < 0.0001)	Not mentioned	Not mentioned	Martínez et al. 2020 [66]
MFG-E8	ALS: 19 Control: 15	sandwich ELISA kit	Anti-inflammatory	MFG-E8 level increased 2503.70 (1294.79) pg/ml compared to normal participants 1332.23 (910.37) pg/ml	Not mentioned	0.77	Yang et al. 2020 [65]
NFL	ALS: 19 Control: 15	sandwich ELISA kit	Axonal transport, shaping cells	NFL level increased 151.12 (9.88) pg/ml compared to normal participants 140.90 (12.85) pg/ml	Not mentioned	0.77	Yang et al. 2020 [65]
PGRN	ALS: 16 Control: 17	human PGRN ELISA kit	Inflammation	The PGRN level was 4.1 ± 1.5 ng in ALS compared to 4.0 ± 1.1 ng in normal participants	Not mentioned	Not mentioned	Feneberg et al. 2016 [62]
CD14	ALS: 40 Control: 19	R&D Systems® ELISA Kits	Inflammatory markers secreted from Hepatocytes	Elevated in ALS comparted to normal participants (ALS mean ~ 150ng/mL, normal ~ 110ng/mL)	Not mentioned	Not mentioned	Beers DR et al. 2020 [68]
Exosomes biomarkers							
miR-124-3p (exosomes)	ALS: 6 Control: 9	Total Exosome Isolation Reagent (#4,484,453, Thermo Fisher Scientific)	Anti-inflammatory	Elevated levels in ALS patients than controls. a significant association between exosomal miR-124-3p levels and ALS disease clinical stage	Not mentioned	Not mentioned	Yelick et al. 2020 [86]
CUEDC2 (exosomal mRNA)	ALS: 4 Control: 4	exoRNeasy Serum/Plasma Midi Kit (QIAGEN, Hilden, Germany) Agencourt RNAdvance Tissue Kit (Beckman Coulter, CA, USA)	Correlated with oxidative stress response, unfolded protein response	Upregulated in patients with ALS. Potential candidate for ALS-related disease biomarker	Not mentioned	Not mentioned	Otake et al. 2019 [87]

The C9orf72 repeat expansion GGGGCC (G_4_C_2_)n is the most common genetic cause of FTLD and 25–40% of all FALS cases [7, 10, 12, 15]. C9orf72 encodes five dipeptide repeat proteins (DPRs) observed in patients with ALS and FTLD [10], exhibiting neurotoxic effects. These proteins have been detected in CSF and blood and thus could be a valuable fluid-based biomarker tracking ALS pathology [59] (Fig. 2).

Due to recent proteomic findings, inflammatory factors were identified to be altered in the CSF of patients with ALS, reflecting the potential role of inflammatory mediators as biomarkers of ALS. For instance, elevated levels of interleukin-6 (IL-6) in astrocyte-derived exosomes of SALS patients have been identified, showing a positive correlation with the rate of disease progression [8, 10, 60]. Progranulin (PGRN), a secretory protein involved in inflammation and other cellular functions, is another mediator that changes in CSF of FTD, ALS, and AD patients. Multiple studies detected higher cerebrospinal fluid PGRN levels in ALS-diagnosed patients [61–63]. Moreover, milk fat globule-EGF factor 8 (MFG-E8), a secretory glycoprotein with anti-inflammatory properties [64], is another mediator involved in the pathogenesis of neurodegenerative diseases, including ALS. It has been found that MFG-E8 plays a role in distinguishing ALS from healthy controls, as ALS patients exhibit a higher CSF level of MFG-E8 [64, 65]. As some papers report, macrophage chemoattractant protein-1 (MCP-1) and macrophage inflammatory protein-1β (MIP-1β) levels significantly increased in the CSF fluid of ALS patients compared to the healthy controls [54, 66, 67] (Table 2). In one cohort study, these cytokine levels were analyzed concerning ALS duration and severity, as well as its pathogenesis. Although these cytokines affected ALS pathogenesis, no correlation with the duration or severity of the disease was found [66] (Fig. 3). Besides, the enhanced level of CD14, secreted from hepatocytes, in the ALS group could be detected in the CSF and serum of the patients [68, 69].

Lastly, blood metabolic parameters can also be examined as potential biomarkers in diagnosing ALS. A metabolomics study of plasma approved by the Institutional Review Board at Massachusetts General Hospital identified a 32-member panel of metabolites that differentiated patients with ALS from healthy controls [70]. This biomarker panel included creatinine, creatine, glutamine, pyroglutamine, and urate. Other studies have demonstrated that uric acid level in ALS patients was significantly lower [8, 48, 62], similar to serum albumin level in those patients [8, 54]. Furthermore, creatinine level in serum was observed to be increased in the early stages of ALS [71] but decreased along the progression of the disease [72–74]. Nonetheless, additional investigations might be needed to confirm the utility and specificity of these biomarkers in the ALS diagnosis [10, 70] (Fig. 3).

## Discussion

While many studies elaborate on the research aspect of the biomarkers and report all potential biomarkers, our study only includes the biomarkers and detecting kits that currently could be measured in rural laboratories without implementing high-tech instruments. Moreover, unlike similar articles, we reported commercial kits of the ALS detecting disease. Therefore, identifying and measuring ALS biomarkers is critical for accurate and rapid diagnosis at the early stages, helping physicians and caregivers handle the situation most effectively [75].

The problem in finding potential biomarker arise in the specificity aspect, in which almost all of the reported biomarkers are common between two or more diseases. Implementing specific instruments, including neuroimaging and electromyography (EMG) in differentiated diagnostics of ALS mainly has a doubtful result, which leads the physician to roll out other diseases rather than directly justify ALS. Researchers mainly perform neuroimaging techniques listed as T1 weighted, diffusion tensor imaging (DTI), and magnetic resonance spectroscopy (MRS) to diagnose ALS. In the T1 weighted images, multiple areas are claimed to be changed as a result of ALS disease bulbar onset. Among them, hypothalamus atrophy is more prominent compared to the control group [76]. Nevertheless, the issue of specificity arises due to the same reduction observed in the autism spectrum [77]. The thickness of the temporal muscle is reported to be altered in the ALS group. Besides its novelty, the temporalis muscle is susceptible to several diseases such as glioblastoma [78]. To evaluate the DTI method, Agosta et al. systematic review could be referred to, in which five studies reported decreased FA parameter as a biomarker in the corpus callosum (CC) [79]. Likewise, in AD at the mild stage, the FA value decreased significantly in the CC [80]. Finally, the value of the diagnosis of the MRS was assessed in a systematic review of six studies, in which it could be observed a decrease in the N-Acetyl-aspartate (NAA) concentration. Accordingly, the NAA/creatine (NAA/Cr) ratio and the NAA/choline (NAA/Cho) ratio were diminished [81]. Nevertheless, the decreased ratio of NAA to creatine is observed in multiple sclerosis (MS) and it construes as the fatigue sign of the disease, whereas this ratio in MS is mostly affected by creatine rather than NAA [82]. Another utmost important instrument in ALS detection is known as EMG and nerve conduction study (NCS). The overall evaluation through NCS is concluded by measuring F-wave and compound motor action potential (CMAP). F-waves and CMAP alone do have not a potential diagnostic value in differentiating ALS from other neuromuscular diseases [83]. Alternatively, a scale calculated from the CMAP value of the ulnar and median nerve (Abductor pollicis brevis (APB) CMAP divided by Abductor digiti minimi (ADM) CMAP) named split hand has a high specificity and sensitivity in ALS detection with 81% and 78%, respectively [84]. Among the multiple limitations in performing the mentioned instruments, two of them are more significant in clinical approaches, including case-by-case difference explanations of the data results along with vague cut-off points. ALS-specific biomarkers in the EVs could be a potential candidate for overcoming with mentioned challenges since the coincidence of positive results of a complex set of ALS biomarkers in the EVs has the least chance to be observed in other diseases. Nevertheless, one of the current study limitation was lack of reporting sensitivity and specificity in the included articles reported exosomal biomarkers, in which conducting a comparison between the sensitivity and specificity of the exosomal and non-exosomal biomarkers was not possible.

## Conclusion

In conclusion, due to the heterogeneity of the ALS disease manifestation and onset, finding a single specific fluid-based biomarker for ALS is far-reaching. Alternatively, a combination of biomarkers should be measured. Biomarkers are the indicators of the different cellular pathways involved in disease onset and progression which help to develop effective therapeutics in the future as well as monitor the disease progression. In this regard, diagnosis based on plasma and CSF EVs can provide helpful information about abnormal protein segregations and changes in miRNA levels in the physiological fluids of the human body. To reach this purpose, six commercial kits are listed: ExoQuick, ExoQuick Plus, Exo-spin, ExoEasy, Exo-Flow, and ME kits are beneficial in extracting exosomes from the body fluids [22]. Exosome markers including surface markers (CXCL5, S100A9, CXCL12, CD63, CD9, and TSG101), can be detected by western blot and Luminex assay to justify the accuracy of the exome extracting procedure [22].

## Data Availability

Not applicable.
